# The Glucagon Receptor Is Expressed in the Frontal Cortex and Impaired Signaling Associates With Cognitive Decline

**DOI:** 10.1210/jendso/bvaf056

**Published:** 2025-04-02

**Authors:** Sasha A S Kjeldsen, Jonas Folke, Maud E Ottenheijm, Marie Winther-Sørensen, Jónvá Hentze, Pia Nyeng, Sara L Garcia, Agata Casado-Sainz, Sanne Simone Kaalund, Reidar Albrechtsen, Karina Banasik, Søren Brunak, Nina L Hansen, Jens Juul Holst, Mette M Rosenkilde, Jørgen Rungby, Majken K Jensen, Birgitte Georg, Jens Hannibal, Tomasz Brudek, Susana Aznar, Nicolai J Wewer Albrechtsen

**Affiliations:** Department of Clinical Biochemistry, Copenhagen University Hospital-Bispebjerg and Frederiksberg, Copenhagen 2400, Denmark; Novo Nordisk Center for Protein Research, Faculty of Health and Medical Sciences, University of Copenhagen, Copenhagen 2200, Denmark; Centre for Neuroscience and Stereology, Copenhagen University Hospital-Bispebjerg and Frederiksberg, Copenhagen 2400, Denmark; Copenhagen Centre for Translational Research, Copenhagen University Hospital-Bispebjerg and Frederiksberg, Copenhagen 2400, Denmark; Department of Clinical Biochemistry, Copenhagen University Hospital-Bispebjerg and Frederiksberg, Copenhagen 2400, Denmark; Novo Nordisk Center for Protein Research, Faculty of Health and Medical Sciences, University of Copenhagen, Copenhagen 2200, Denmark; Department of Clinical Biochemistry, Copenhagen University Hospital-Bispebjerg and Frederiksberg, Copenhagen 2400, Denmark; Novo Nordisk Center for Protein Research, Faculty of Health and Medical Sciences, University of Copenhagen, Copenhagen 2200, Denmark; Centre for Neuroscience and Stereology, Copenhagen University Hospital-Bispebjerg and Frederiksberg, Copenhagen 2400, Denmark; Copenhagen Centre for Translational Research, Copenhagen University Hospital-Bispebjerg and Frederiksberg, Copenhagen 2400, Denmark; Department of Science and Environment, Roskilde University, Roskilde 4000, Denmark; Novo Nordisk Center for Protein Research, Faculty of Health and Medical Sciences, University of Copenhagen, Copenhagen 2200, Denmark; Centre for Neuroscience and Stereology, Copenhagen University Hospital-Bispebjerg and Frederiksberg, Copenhagen 2400, Denmark; Copenhagen Centre for Translational Research, Copenhagen University Hospital-Bispebjerg and Frederiksberg, Copenhagen 2400, Denmark; Centre for Neuroscience and Stereology, Copenhagen University Hospital-Bispebjerg and Frederiksberg, Copenhagen 2400, Denmark; Copenhagen Centre for Translational Research, Copenhagen University Hospital-Bispebjerg and Frederiksberg, Copenhagen 2400, Denmark; Biotech Research and Innovation Centre, University of Copenhagen, Copenhagen 2200, Denmark; Novo Nordisk Center for Protein Research, Faculty of Health and Medical Sciences, University of Copenhagen, Copenhagen 2200, Denmark; Novo Nordisk Center for Protein Research, Faculty of Health and Medical Sciences, University of Copenhagen, Copenhagen 2200, Denmark; Department of Clinical Biochemistry, Copenhagen University Hospital-Bispebjerg and Frederiksberg, Copenhagen 2400, Denmark; Department of Biomedical Sciences, Faculty of Health and Medical Sciences, University of Copenhagen, Copenhagen 2200, Denmark; Novo Nordic Foundation Center for Basic Metabolic Research, Faculty of Health and Medical Sciences, University of Copenhagen, Copenhagen 2200, Denmark; Department of Biomedical Sciences, Faculty of Health and Medical Sciences, University of Copenhagen, Copenhagen 2200, Denmark; Steno Diabetes Center Copenhagen, Copenhagen 2730, Denmark; Department of Clinical Medicine, University of Copenhagen, Copenhagen 2200, Denmark; Section of Epidemiology, Department of Public Health, Faculty of Health and Medical Sciences, University of Copenhagen, Copenhagen 1357, Denmark; Department of Clinical Biochemistry, Copenhagen University Hospital-Bispebjerg and Frederiksberg, Copenhagen 2400, Denmark; Department of Clinical Biochemistry, Copenhagen University Hospital-Bispebjerg and Frederiksberg, Copenhagen 2400, Denmark; Department of Clinical Medicine, University of Copenhagen, Copenhagen 2200, Denmark; Centre for Neuroscience and Stereology, Copenhagen University Hospital-Bispebjerg and Frederiksberg, Copenhagen 2400, Denmark; Copenhagen Centre for Translational Research, Copenhagen University Hospital-Bispebjerg and Frederiksberg, Copenhagen 2400, Denmark; Centre for Neuroscience and Stereology, Copenhagen University Hospital-Bispebjerg and Frederiksberg, Copenhagen 2400, Denmark; Copenhagen Centre for Translational Research, Copenhagen University Hospital-Bispebjerg and Frederiksberg, Copenhagen 2400, Denmark; Department of Clinical Biochemistry, Copenhagen University Hospital-Bispebjerg and Frederiksberg, Copenhagen 2400, Denmark; Novo Nordisk Center for Protein Research, Faculty of Health and Medical Sciences, University of Copenhagen, Copenhagen 2200, Denmark; Copenhagen Centre for Translational Research, Copenhagen University Hospital-Bispebjerg and Frederiksberg, Copenhagen 2400, Denmark; Department of Clinical Medicine, University of Copenhagen, Copenhagen 2200, Denmark

**Keywords:** glucagon receptor, postmortem brain, cognitive decline, immunohistochemistry, in situ hybridization, UK Biobank

## Abstract

Individuals with type 2 diabetes (T2D) have an elevated risk of cognitive decline, yet the mechanisms connecting these pathologies remain unclear. Altered glucagon and insulin signaling contribute to T2D, and insulin resistance may also be associated with cognitive decline. The role of glucagon in this context is unknown. Here we aimed to characterize glucagon receptor (GCGR) expression in brain tissue and investigate the potential impact of altered GCGR signaling on dementia prevalence and cognitive function. We investigated GCGR protein expression in various human brain regions and cell types in postmortem brain samples. To explore the potential link between GCGR signaling and cognitive function, individuals with specific GCGR mutations with known or predicted impaired GCGR signaling were examined in connection to the prevalence of dementia defined by International Classification of Diseases, Tenth Revision coding and by cognitive function using population-scale cognitive tests in the UK Biobank. GCGR mRNA and protein were expressed specifically in neurons of the frontal cortex. Varying degrees of expression were observed across brain regions and with higher expression in the parietal cortex and thalamus by antibody-dependent analyses. GCGR variant carriers did not have a significantly higher prevalence of dementia, but 1 cognitive test was significantly impaired in individuals with a GCGR cAMP loss-of-function variant compared to sex- and age-matched nonvariant carrier controls. Our findings indicate GCGR expression in the human brain, particularly in neurons of the frontal cortex, and altered glucagon signaling may be associated with lower cognitive function. Further research is needed to elucidate mechanisms underlying the potential link between altered GCGR signaling and cognitive decline.

Individuals with type 2 diabetes (T2D), characterized by impaired insulin action and enhanced glucagon secretion (hyperglucagonemia), resulting in hyperglycemia [1, 2], have a higher risk of cognitive decline [3-5]. However, the mechanisms that connect these pathologies are unknown. Impaired insulin receptor signaling is a common feature in both T2D and Alzheimer's disease [6-8], and central (brain) insulin resistance may potentially contribute to cognitive decline in individuals with diabetes.

Investigations into central glucagon receptor (GCGR) signaling are limited, but the available data indicate that activation has a potential glucose-lowering effect, which opposes that of hepatic GCGR signaling [9]. Glucagon has previously been associated with neuronal function [10], and a single study has also shown that glucagon provides neuroprotection in mice following a traumatic brain injury [11]. Glucagon is a peptide hormone secreted from pancreatic α cells in response to hypoglycemia or protein intake. Glucagon signals through its cognate G protein-coupled receptor, the GCGR, mainly located in hepatocytes. Glucagon may also cross the blood-brain barrier [12], and the extraction rate of glucagon may even be higher across the brain compared to the liver [13]. The GCGR has been reported in hypothalamic nuclei in rats [14], but a thorough mapping of the GCGR in the human brain and its potential association with cognitive decline has not previously been investigated.

Here, we investigated GCGR localization in the human brain, focusing on its specific localization in cell types of the frontal cortex and putamen using antibody-dependent analyses. We aimed to investigate the potential impact of lowered GCGR signaling on, specifically, cognitive functions and therefore further validated our findings of GCGR expression in the frontal cortex using antibody-independent analyses. The potential association between GCGR signaling and cognitive functions was explored by the assessment of dementia prevalence as identified by International Classification of Diseases, Tenth Revision (ICD-10) coding and cognitive decline as assessed by population-scale cognitive tests in individuals with impaired GCGR signaling identified by specific GCGR loss-of-function (LoF) mutations [15]. We hypothesized that lowered GCGR signaling would impair cognitive function.

## Materials and Methods

### Brain Tissue Samples

Tissue from 4 old but healthy human brains were included in the immunohistochemical and Western blotting experiments. Brains were generously donated to the Bispebjerg Brain Bank, Centre for Neuroscience and Stereology, Department of Neurology, Copenhagen University Hospital Bispebjerg, Denmark. The project was approved by the Danish Data Protection Agency (BFH-2017-001, p-2023-15234), and the Regional Ethical Committee, Capital Region Denmark (H-23005229). A detailed neuropathological histological examination for the presence of α-synuclein, amyloid-β, and tau accumulation was conducted on all brains (Table 1). Braak tau tangle stages ranged from 0 to 3, while no amyloid-β or α-synuclein pathology was observed. We also included tissue from the adenohypophysis and neurohypophysis from C56B mice as positive and negative controls, respectively, based on a prior study from our group [16].

**Table 1.** Patient characteristics at the time of death

Brain	#1	#2	#3	#4
Age	91	100	82	97
Sex	F	M	M	F
Brain weight (g)	1192	1192	1126	1160
Body weight (kg)	50	NA	91	56
Height (cm)	155	NA	174	160
BMI (kg/m^2^)	20.9	NA	30.1	21.9
Hemisphere	Left	Right	Right	Right
Braak stage (tau)	0	3	3	1
Postmortem interval (h)	24	24	48	90

Brains were donated to the Bispebjerg Brain Bank at Copenhagen University Hospital Bispebjerg and Frederiksberg.

Abbreviations: BMI, body mass index; NA, not available.

Brains are stored with 1 hemisphere frozen at −80 °C and the other hemisphere in formalin. Tissues for Western blotting experiments were dissected from frontal cortex [Brodmann area (BA) 46], temporal cortex (BA 21/22), parietal cortex (BA 7), occipital cortex (BA 17/18), thalamus, hypothalamus, anterior cerebellar cortex, putamen, medulla, and pons areas from the frozen hemisphere. Paraffin-embedded formalin-fixed tissue blocks dissected from the frontal cortex (BA 46) and putamen from 3 brains were used (brain #1-#3 in Table 1) for immunohistochemistry (using either a conjugating enzyme or a fluorophore as the quantifiable measure) and in situ hybridization. The same GCGR antibody (cat# ab75240, lot# GR2260811-14, RRID: AB_1523687, Abcam) was used for all the Western blotting and immunohistochemical experiments. The GCGR antibody has been extensively validated [17]. Key resources are provided in Supplementary Table S1 [18].

### Western Blotting Procedures

#### Preparation of brain tissue

For Western blotting, approximately 50 mg frozen tissue was cut out from the regions of interest and stored at −80 °C. To the tissue was added 500 µL of tissue extraction reagent II (cat# FNN0081, Thermo Fisher Scientific) with 1% protease inhibitor cocktail (cat# P8340, Sigma-Aldrich) in Magna Lyser Green beads (cat# 03358941001, Roche Diagnostics), and it was homogenized twice (25 seconds at 6000 RPM) with a Magna Lyser instrument (Roche Diagnostics) followed by 90 seconds on a MagNA Lyser Rotor Cooling Block (cat# 03359085001, Roche Life Science). For the adeno- and neurohypophysis, only 50 µL of lysis master mix was used. Samples were centrifuged for 1 minute (16 000G at 4 °C) to reduce foam and transferred to Eppendorf tubes. After 20 minutes of centrifugation (16 000G at 4 °C), supernatant was transferred to fresh Eppendorf tubes and then aliquoted and stored at −80 °C. Total protein content was measured on a 1:4 diluted sample using Bradford Assay (cat# B6916, Sigma-Aldrich) and read at 620 nm on a Multiskan FC Microplate reader (cat# 51119000, Thermo Fisher Scientific). Human embryonic kidney (HEK)-293 cells transfected with human GCGR or mock-transfected (previously described in [17]) were extracted using a subcellular protein fractionation kit (cat# 78840, Thermo Fisher Scientific) and used as positive and negative controls, respectively.

#### Western blotting

Brain protein extract containing 10 µg of protein was mixed with 4× loading buffer (cat# 928-4004, Li-Cor) and 20% of 0.1 M dithiothreitol, then boiled at 70 °C for 10 minutes. The HEK-293 cells transfected with the GCGR (positive control) or mock-transfected (negative control) were loaded as 5 µg and 1 µg protein. Samples and ladder (molecular weight marker) (Chameleon Duo prestained ladder, cat# 928-60000, Li-Cor) were separated on NuPAGE 4-12% Bis-Tris gels (cat# NP0329BOX, Invitrogen) at 200 V for 30 minutes, with NuPAGE MES SDS Running Buffer (1:20, cat# NP0002, Life Technologies) and NuPAGE Antioxidant (1:400, cat# NP0005, Invitrogen) in PBS and subsequently transferred onto iBlot2 nitrocellulose membranes (cat# IB23002, Invitrogen) using the iBlot2 Gel Transfer Device (Invitrogen). Total protein staining was done using the REVERT 700 Total Protein Stain protocol (cat# 926-11021, Li-Cor) and imaged at 700 nm for 30 seconds with the LiCor Odyssey FC Imaging system and ImageStudio v4.0. Membranes were then blocked with blocking buffer (cat# 927-70001, Li-Cor) for 1 hour at room temperature and incubated overnight at 4 °C with rabbit anti-human GCGR polyclonal primary antibody (1:1000, cat# ab75240, lot# GR2260811-14, RRID: AB_1523687, Abcam). On day 2, membranes were washed 3 times in PBS containing 0.1% Tween-20 (cat# 9005-64-5, Merck) and incubated for 1 hour at room temperature with anti-rabbit IgG secondary antibody (1:15.000, cat# 926-32211, RRID: AB_621843, Li-Cor) in a buffer of PBS containing 0.1% Tween-20 and 0.01% SDS. Membranes were then washed (3 times with PBS) and air dried under a dark cover, then subsequently imaged with the Li-Cor Odyssey FC Imaging system and ImageStudio v4.0 at 700 and 800 nm for 2 minutes each. Blot image intensities were measured at approximately 55 kDa using ImageStudio v4.0 image analysis tools. Band intensities were adjusted to corresponding total protein band intensities and the positive control band to account for both loading and interblot variability.

### Immunohistochemistry

#### Preparation of brain tissue

Blocks of brain tissue (∼1-2 cm^3^) from the frozen hemisphere were dissected from the fresh brains and fixed in 10% buffered formalin (cat# 1000.5000, CellPath) for a minimum of 48 hours and subsequently embedded in paraffin on a Leica ASP300S tissue processor (Leica Biosystems) and stored at room temperature until sectioning. Tissue was cut in 10 µm sections on a Leica RM2125 RTS microtome (Leica Biosystems) and placed on Starfrost glass slides (cat# 2510.1250, Cardinal Health). Slides were heated at 60 °C for 40 minutes and stored at room temperature until staining.

#### Immunohistochemistry

Sections were deparaffinized by washing in xylene (cat# 02080, Histolab) for 2 × 5 minutes, 99% ethanol for 2 × 2.5 minutes (cat# 720915, Apoteket Region Hovedstaden), 96% ethanol for 2 minutes (cat# 720907, Apoteket Region Hovedstaden), 70% ethanol for 2 minutes (cat# 720883, Apoteket Region Hovedstaden), and running tap water for 5 minutes. They were heated in citrate buffer (pH 6, cat# 850180, Apoteket Region Hovedstaden) for ∼20 minutes in a microwave for antigen retrieval and cooled in the buffer for 20 minutes at room temperature. This was followed by 2 washing steps in PBS and subsequent overnight incubation at 4 °C with rabbit anti-human GCGR polyclonal primary antibody (1:200, cat# ab75240, lot# GR2260811-14, RRID: AB_1523687, Abcam) diluted in PBS. The next day, samples were washed twice with PBS and blocked with 3% H_2_O_2_ for 8 minutes. Sections were incubated with goat anti-rabbit HRP-labeled EnVision + complex (cat# K4003, lot# 11478723, RRID: AB_2630375, DAKO) for 30 minutes at room temperature, washed twice in PBS, and developed with the 3,3′-diaminobenzidine Bright system (cat# VWEKBS04-500, ImmunoLogic) for 8 minutes. After washing with dH_2_O, nuclear staining with Mayer's hematoxylin (cat# 860213, Apoteket Region Hovedstaden) was done for 30 to 45 seconds, followed by a 10-minute wash with running tap water and further dehydration with ethanol. Lastly, sections were mounted with Pertex mounting medium (cat# 00801, Histolab) and dried overnight at room temperature before being examined using an Olympus BX50 light microscope at 40× and 60× magnification.

#### Double/triple immunofluorescence

For immunofluorescence, the same procedure was carried out for deparaffinization and antigen retrieval. Cells were subsequently permeabilized in 0.25% Triton X-100 (cat# 9036-19-5, Sigma-Aldrich) in PBS for 3 × 5 minutes, and autofluorescence was quenched with 0.1% Sudan Black B (cat# 4197-25-5, Sigma-Aldrich) in 70% ethanol (cat# 720883, Apoteket Region Hovedstaden) at room temperature for 30 minutes. Next, samples were washed in a 0.5% Tween-20 (cat# 9005-64-5, Merck) solution in PBS and blocked for 1 hour at room temperature with 10% fetal calf serum (FCS) in PBS. Slides were incubated overnight at 4 °C with rabbit anti-human GCGR polyclonal primary antibody (1:100, cat# ab75240, lot# GR2260811-14, RRID:AB_1523687, Abcam) combined with either mouse anti-neuronal nuclear antigen (NeuN) (1:250, cat# MAB377, RRID: AB_2298772, Chemicon), mouse anti-glial fibrillary acidic protein (GFAP) (1:100, cat# M0761, RRID: AB_2109952, DAKO) or mouse anti-ionized calcium binding adaptor molecule 1 (Iba1) (1:100, Alexa555-conjugated, cat#MABN92-AF555, RRID: AB_10917271, Millipore) diluted in 10% FCS in PBS. The next day, samples were washed in PBS for 3 × 5 minutes. GCGR + NeuN and GCGR + GFAP samples were incubated for 1 hour at room temperature with donkey anti-mouse (1:300, Alexa488-conjugated, cat# 715-545-150, RRID: AB_2340846, Jackson ImmunoResearch) and goat anti-rabbit (1:1000, Alexa594-conjugated, cat# A11012, RRID: AB_2534079, Invitrogen) in 10% FCS in PBS while covered. GCGR + Iba1 samples were first incubated with anti-GCGR antibody overnight at 4 °C, followed by 1 hour incubation with donkey anti-rabbit (1:300, Alexa488-conjugated, cat# 711-545-152, RRID: AB_2313584, Jackson ImmunoResearch) in 10% FCS in PBS while covered. Hereafter, the GCGR + Iba1 samples were incubated overnight at 4 °C with anti-Iba1 antibody (1:100, Alexa555-conjugated, cat# MABN92-AF555, RRID: AB_10917271, Millipore). All samples were then washed in PBS 3 × 5 minutes while shielded from light. Finally, sections were mounted with fluoroshield mounting medium (cat# ab104139, Abcam) and stored in the dark at 4 °C until imaging. Z-stack images were captured using 40 × (UPLSAPO NA 0.95) and 60 × (UPLSAPO oil immersion NA 1.35) objectives of an Olympus LSM FV1200S-IX83 confocal microscope. The image resolution was 1024 × 1024, and the voxel size was 0.31 × 0.31 × 1.16 µm. Laser and detector settings were identical for samples treated with the same antibodies. Z-slices were stacked using the maximum intensity Z projection function (projection type = max intensity) in Fiji to create a single composite image [19].

#### Fluorescence in situ hybridization histochemistry of the GCGR

For detection of glucagon receptor mRNA, a plasmid (MC203290) containing the mGcgr cDNA was obtained from Origen. After expansion, the full coding sequence (nt. 115-1575, BC057988) was amplified by PCR using Pfu polymerase and the following primers mGcgr_Forward: TGTAGAATTCATGCCCCTCACCCAGCTC and mGcgr_Rerverse: TTCAGAATTCAGGTGGGGCTGTCAGCCAA. The obtained PCR fragment was inserted in the EcoRI site of pBluscriptKS+, and the resulting plasmid mGcgrKS + (1.3) was sequence verified. mGcgrKS + (1.3) linearized with either XhoI or XbaI was used as a template for the antisense and sense probe, respectively, and RNA labeled with Dig-11-UTP were made using T7-polymerase (antisense) and T3-polymerase (sense). Fluorescence in situ hybridization using the digoxigenin-labeled antisense and sense probes was performed on sections from the human cortex as previously described [20]. Briefly explained, after hybridization Dig-11-UTP labeled probes were visualized using a sheep anti-digoxigenin peroxidase-conjugated antibody (1:50, POD-conjugated, cat# 11207733910, RRID: AB_514500, Roche) followed by washing and incubation in Alexa Fluor 488-conjugated tyramide (Molecular Probes, diluted 1:100). After washing, slides were mounted in glycerol 4',6-diamidino-2-phenylindole solution (DAPI).

### The UK Biobank

Data were extracted from the large-scale biomedical database UK Biobank in February 2024, which currently includes data from >500 000 individuals recruited throughout the United Kingdom. The study was conducted as part of a larger project (application ID 61785) focusing on genetic variants of the GCGR. For the purposes of this study, whole-exome sequencing data, age, sex, ICD-10-based diagnoses (field #41270), and cognitive test scores were used. All data were filtered to exclude non-Caucasian individuals (field #22006) and all cases of sex chromosome aneuploidy (field #22019). Whole-exome sequencing data were reference-aligned using Original Quality Functional Equivalent sequencing data (field #23141) as previously described [21]. Variant call was performed using DeepVariant [22]. Genomic VCF files were filtered for each sample, restricted to the location of the glucagon receptor at chr17: 81 804 150 to 81 814 008 forward strand. Whole-exome sequence analysis was performed on the UK Biobank's research analysis platform DNA Nexus (https://ukbiobank.dnanexus.com) Output files for the included individuals were processed and merged using Python. Scripts are available at https://github.com/nicwin98/UK-Biobank-GCG.

#### GCGR variant selection

We selected 5 genetic variants of the GCGR that cause a decrease in cAMP accumulation (cAMP LoF: V368M, R378C, R225H, R308W, D63N) based on a previous study that evaluated 38 GCGR missense variants [15]. All individuals were heterozygous carriers. In a separate group, G40S, we pooled individuals heterozygous or homozygous for the G40S allele of the GCGR, shown to impair β-arrestin-1 recruitment [15]. Finally, we included a group of individuals with a frameshift variant of the GCGR for predicted LoF alleles (defined in [21]). The frameshift variants of the GCGR remain to be characterized regarding their GCGR activity. For each GCGR variant group (cAMP LoF, G40S, or frameshift), we investigated the prevalence of all-cause dementia (including Alzheimer's disease and subtypes of dementia) compared to nonvariant carrier controls. In addition, we compared cognitive test results between each GCGR variant group to matched control groups. The matched control groups consisted of nonvariant carriers and were matched on age and sex in a 1:5 ratio using the MatchIt package in R [23].

#### ICD-10-based diagnosis of all-cause dementia

The stratification of all-cause dementia and subtypes (including vascular dementia and Alzheimer's disease) was based on the ICD-10 classification system and comprised A81.0, F00.0, F00.1, F00.2, F00.9, F01.0, F01.1, F01.2, F01.3, F01.8, F01.9, F02.0, F02.1, F02.2, F02.3, F02.4, F02.8, F03, F05.1, F10.6, G30.0, G30.1, G30.8, G30.9, G31.0, G31.1, G31.8, and I67.3 based on a previous study investigating dementia and cognitive decline [24]. The data were summarized in a binary variable to define individuals with or without cumulative all-cause dementia registered from 1996 to November 2023. All-cause dementia prevalence was assessed in individuals with a GCGR variant (cAMP LoF, G40S, and frameshift) and compared to nonvariant carrier controls in November 2023 (termed follow-up).

#### ICD-10-based diagnosis of T2D

The T2D diagnosis was classified similarly to a previous study [25]. Initially, an ICD-10 diagnosis of E11 or E14 was defined as T2D. However, the T2D diagnosis was removed if the participant only had gestational diabetes or if the participant had the ICD-10 code for both type 1 diabetes and T2D and their age at diagnosis was <20 years or they had started insulin therapy within 1 year of diagnosis. Furthermore, participants were classified as T2D if they had the type 1 diabetes diagnosis (classified by ICD-10 coding) and were prescribed oral T2D medication (excluding metformin) or if they had a hemoglobin A1c > 48 mmol/mol and did not have the ICD-10 coding for diabetes. The ICD-10 classification for T2D was used to assess T2D prevalence in individuals with or without the GCGR variants.

#### The assessment of cognitive function

We employed data from self-administered and computerized tests with a touchscreen interface to evaluate cognitive function. These tests were specifically developed for the UK Biobank as they enabled cognitive testing on a population scale (completed by >100 000 individuals) and were completed at the time of participant inclusion (2006-2010) (termed baseline). The Cronbach α coefficient has previously been assessed for the reaction time test as 0.85 [26].

##### Reaction time

The reaction time test was a game in which participants were to press a button as quickly as possible when 2 on-screen cards matched. The score from this task was the mean response time in milliseconds across all trials. A higher score was an indication of slower reaction time.

##### Visual memory test

In the visual memory test, participants viewed a display of cards for a duration of 5 seconds and were subsequently required to recall which cards matched when the cards were no longer visible. The visual memory test is labeled pairs matching in the UK Biobank (http://biobank.ctsu.ox.ac.uk/crystal/label.cgi?id=100030v). The test consisted of 2 trials (arrays). In the first trial, 6 cards (comprising 3 pairs) were shown. In the second trial, 12 cards (comprising 6 pairs) were shown. Similar to a previous study [27], we only used the test score from the second trial (12 cards) as the first trial was deemed too easy. The score for each trial was the number of errors made before matching all pairs. Therefore, higher scores reflect poorer cognitive function.

### Statistical Analysis

Data distribution was assessed by histograms and QQ plots. A Wilcoxon rank sum test with continuity correction was used to compare data, which were nonnormally distributed. All comparator groups in the UK Biobank (cAMP LoF, G40S, and frameshift, respectively) were matched on age and sex in a 1:5 ratio using the Matchit package in R.

We performed logarithmic regression followed by the calculation of odds ratio (OR) using the function glm and oddsratio in R to assess the risk of all-cause dementia or Alzheimer's disease (by ICD-10 classification) in individuals with or without a GCGR missense (cAMP LoF or G40S) or frameshift variant.

Data in the text are presented as mean ± SD if not otherwise indicated. Nonnormally distributed data are presented as median with the first and third quartile.

## Results

Four human brains were assessed postmortem for the evaluation of GCGR expression in different regions. Notably, the fourth donor (#4 in Table 1) had a postmortem interval of 90 hours, which may affect epitope preservation. All 4 brains were used in the Western blotting analyses, but only 3 brains (#1-#3 in Table 1) were used for the immunohistochemical staining and in situ hybridization. The donors were 93 ± 8 years and had a brain weight of 1170 ± 38 grams and a body mass index of 24 ± 5 kg/m^2^ at the time of death (Table 1).

### The GCGR Is Expressed With Varying Degrees in Different Regions of the Brain and Colocalizes With Neurons

**Figure 1.**
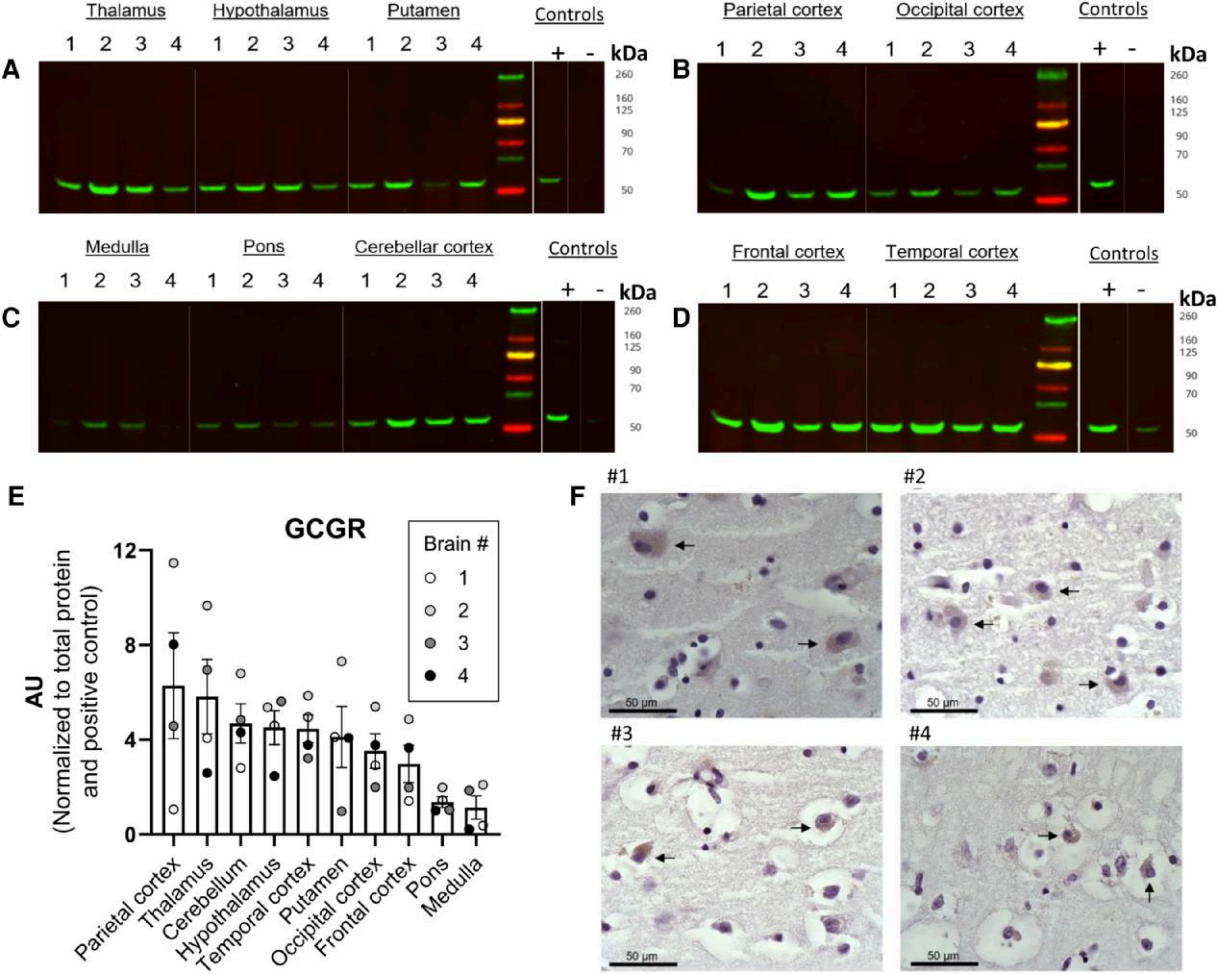
The GCGR is expressed in several human brain regions. Ten different brain regions were investigated for GCGR protein expression using Western blotting technique with a previously validated GCGR antibody (cat# ab75240, lot# GR2260811-14, RRID: AB_1523687, Abcam). (A-D) Four cropped blots for GCGR expression (55 kDa) are shown with corresponding positive (GCGR-transfected HEK-293 cells) and negative (mock-transfected HEK-293 cells) controls. Uncropped blots are shown in Supplementary Fig. S1 [18]. Control samples were loaded (1 µg protein) on each blot. Brain region names and brain numbers are shown above (A + B) or below (C + D) the corresponding band for each blot. (E) Protein levels of the GCGR in brain tissue regions (n = 4, loaded with 10 µg protein) measured by Western blotting. Levels of the GCGR were normalized to the total protein concentration for the individual blot and subsequently to the positive control to adjust for interblot variation. (F) Immunohistochemistry staining for the GCGR in the frontal cortex. #1 to #4 correspond to brain #1 to #4 in Table 1. Data are presented as means ± SEM. Abbreviations: AU, arbitrary units; GCGR, glucagon receptor; HEK, human embryonic kidney; TP, total protein.

**Figure 2.**
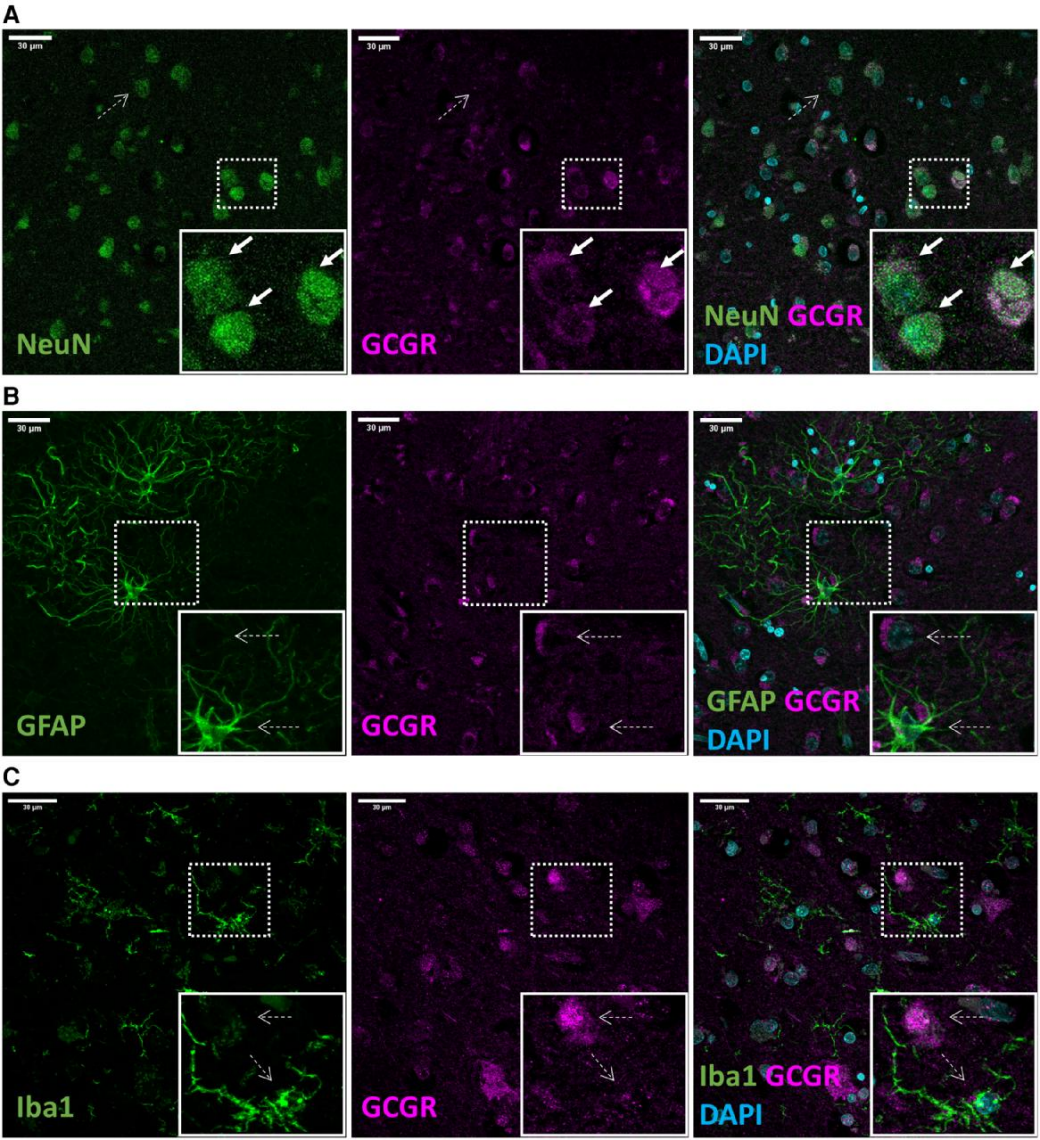
Immunofluorescent staining shows GCGR positivity in neurons of the human frontal cortex. Representative images from 10 µm sections of a 1 to 2 cm^3^ tissue block of frontal cortex from 3 human brains (#1-#3) stained with (A) mouse anti-NeuN (staining for neurons) and rabbit anti-GCGR (brain #3), (B) mouse anti-GFAP (staining for astrocytes) and rabbit anti-GCGR (brain #1), or (C) mouse anti-Iba1 (staining for microglia) conjugated to Alexa Fluor 555 and rabbit anti-GCGR (brain #2). All sections were also stained with anti-mouse Alexa488 and anti-rabbit Alexa594 or anti-rabbit Alexa488 as a control for nonspecific binding of the secondary antibody and DAPI (cyan, cell nuclei) (Supplementary Fig. S2) [18]. Examples of positive colocalization are indicated by the arrows and no colocalization by stippled arrows. The dashed square in each panel highlights the region that is magnified in the corresponding inset located in the lower right corner of the panel. An overview of the employed antibodies is presented in Supplementary Table S1 [18]. Scale bars: 30 µm. Abbreviations: DAPI, 4',6-diamidino-2-phenylindole; GCGR, glucagon receptor; GFAP, glial fibrillary acidic protein; Iba1, ionized calcium binding adaptor molecule 1; NeuN, neuronal nuclear antigen.

We performed Western blotting analysis to investigate protein expression of the GCGR in 10 different regions of the brain and compare GCGR protein abundance within these regions (Fig. 1A-1D). Band expression was shown at the predicted size of the GCGR, and band intensity quantification revealed that the GCGR is expressed with varying degrees in various regions of the brain (Fig. 1E) with the highest relative protein expression in the parietal cortex and thalamus and the lowest protein expression in the pons and medulla of the hindbrain. Uncropped blots are presented in the Supplementary Material [18]. Positive controls (HEK-293 cells transfected with the human GCGR) revealed detectable GCGR expression with both 1 and 5 ug protein loading. We observed positive GCGR expression with the 5 ug but not the 1 ug loading of the negative controls (mock-transfected HEK-293 cells), indicative of low levels of endogenous GCGR expression in HEK-293 cells (Supplementary Fig. S1) [18]. As expected, we found a positive band in tissue from the adenohypophysis (positive control) but not the neurohypophysis (Supplementary Fig. S2) [18]. We then performed immunohistochemical staining of the corresponding frontal cortex (from the same individuals we performed Western blotting on), revealing cytoplasmic staining consistent with the antibody epitope of the C-terminus of the GCGR (Fig. 1F). To identify the cell types that express the GCGR, we conducted immunofluorescent double-staining experiments. For this purpose, sections of the frontal cortex and putamen of 3 human brains (#1-#3 in Table 1) were stained for the GCGR and cell markers for neurons (NeuN), astrocytes (GFAP), and microglia (Iba1). The double staining with anti-NeuN and anti-GCGR antibodies showed positive expression of the GCGR in NeuN-positive neurons (Fig. 2A) in the frontal cortex. The NeuN marker also stained blood vessels. The GCGR was not expressed in astrocytes (GFAP) or microglia (Iba1) (Fig. 2B and 2C). Similar results were found when staining sections of the putamen (Supplementary Fig. S3) [18].

Two of the 4 brains had Braak stage 3, which indicates a moderate stage of neurofibrillary tangle development in the brain, not unusual for the age of the donors. Upon visual examination, the abundance of GCGR expression did not seem to be dependent on Braak stage.

**Figure 3.**
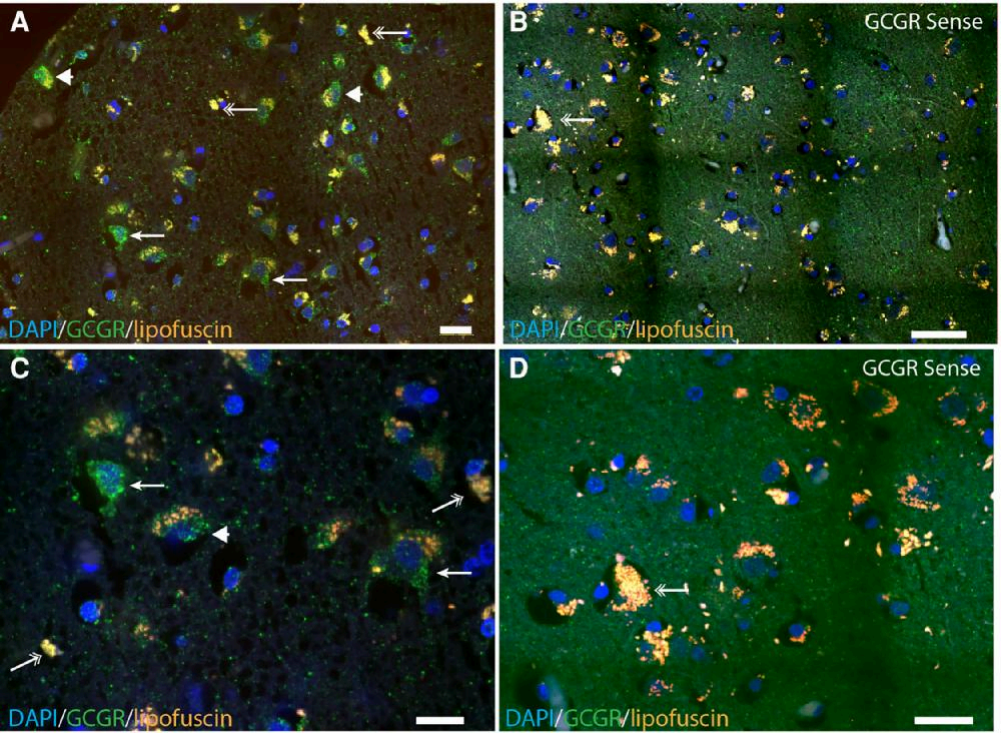
Fluorescence in situ hybridization histochemistry demonstrates GCGR mRNA in neurons of the frontal cortex. Representative images from 10 µm sections of a 1 to 2 cm^3^ tissue block of frontal cortex from human brains demonstrate GCGR mRNA (green) in A and C using anti-sense probes labeled with digoxigenin and visualized by Alex488-tymamide. Single arrows indicate neurons without lipofuscin (autofluorescence); arrowheads indicate neurons expressing the GCGR mRNA and containing lipofuscin granula. Double arrows indicate GCGR mRNA-negative cells containing lipofuscin granula. Nuclei are counterstained by DAPI (blue). (B) and (D) show the sense-hybridization control sections displaying only lipofuscin granula-containing cells. Scale bars: 15 µm. Abbreviation: GCGR, glucagon receptor.

To verify the presence of GCGR protein in neuronal tissue, we used the antibody-independent method fluorescence in situ hybridization. GCGR mRNA was located in neurons of the frontal cortex, and no signals were found using the negative control (Fig. 3).

### All-cause Dementia and Cognitive Test Scores in Individuals With Impaired GCGR Signaling in UK Biobank

We aimed to explore whether changes in GCGR signaling, induced by genetic alterations in the gene that codes for the GCGR, are associated with to cognitive decline or an increased prevalence of all-cause dementia in humans. GCGR variant carriers were stratified into 3 groups: (1) individuals with a GCGR missense variant(s) that reduce the production of cAMP (cAMP LoF), as demonstrated previously in vitro [15]; (2) hetero- and homozygous carriers of the G40S allele of the GCGR, which impairs β-arrestin-1 recruitment [15]; and (3) individuals with a GCGR frameshift mutation (not yet pharmacologically characterized for GCGR activity) (Supplementary Table S2) [18]. Three participants were classified as having both a cAMP LoF variant and a G40S variant and were included in both subgroups.

### Cognitive function in GCGR variant carriers

First, we aimed at functionally assessing cognitive ability by population-scale cognitive tests in individuals with or without the 3 types of GCGR variants. All individuals without missing values for age, sex, and the cognitive tests for reaction time and visual memory at study inclusion were included in the analyses. Descriptive data for the whole cohort are presented in Table 2, of which 385 343 nonvariant carrier controls, 109 individuals with a cAMP LoF variant, 6733 individuals with a G40S allele, and 240 individuals with a frameshift mutation had completed both tests. Cognitive test scores were assessed for each GCGR variant carrier group and statistically compared with nonvariant carrier age- and sex-matched controls.

**Table 2.** Descriptive data for individuals with a GCGR variant (cAMP LoF, G40S, or frameshift) and nonvariant carrier controls at study inclusion (baseline)

Participant characteristics at baseline	Nonvariant carrier controls	cAMP LoF variant	G40S variant	Frameshift variant
n	385 343	109	6733	240
Age	58 (51, 63)	57 (49, 62)	58 (51, 63)	59 (52, 65)
Sex (female), %	54.1	59.6	55.0	57.9
T2D (yes), %	3.9	1.8	4.0	2.0
Reaction time, ms	535 (480, 606)	539 (496, 598)	536 (480, 610)	543 (481, 597)
Visual memory (pairs matching), no. of errors	277 (215, 361)	294 (228, 382)	276 (214, 363)	280 (220, 371)

Three participants are presented in both the cAMP LoF variant column and the G40S variant column as they had both GCGR variants. Only participants with no missing values for age, sex, and cognitive test scores for “reaction time” and “visual memory” are presented. A higher test score is indicative of lower cognitive function. Data are presented as median (first quartile, third quartile).

Abbreviations: GCGR, glucagon receptor; LoF, loss-of-function; T2D, type 2 diabetes.

Individuals with a GCGR cAMP LoF vairant had a significantly higher visual memory test score (*P* = .02), indicating diminished cognitive function, compare to age- and sex-matched nonvariant carrier controls (Table 3). The reaction time test did not elicit a significant difference between groups. The prevalence of T2D was 1.8% in individuals with the cAMP LoF GCGR variant and 3.5% in age- and sex-matched nonvariant carrier controls. Hetero- and homozygous carriers of the G40S allele and individuals with the frameshift variant did not have diminished cognitive function (Table 3).

**Table 3.** Cognitive tests (at baseline) between individuals with a GCGR variant (cAMP LoF, G40S, or frameshift variant) and age- and sex-matched nonvariant carrier controls

	cAMP LoF			G40S			Frameshift		
Variable	Age- and sex-matched controls	cAMP LoF carriers	*P*	Age- and sex-matched controls	G40S carriers	*P*	Age- and sex-matched controls	Frameshift carriers	*P*
Age	57 (49, 62)	57 (49, 62)		58 (50, 63)	58 (50, 63)		59 (51, 65)	59 (51, 65)	
Females, %	59.6	59.6		54.9	54.9		57.9	57.9	
T2D, %	3.5	1.8		4.0	4.0		4.3	2.1	
Reaction time, ms	531 (476, 598)	539 (496, 598)	.11	535 (480, 606)	536 (480, 610)	.14	532 (481, 601)	543 (481, 597)	.69
Visual memory, no. of errors	265 (208, 352)	294 (228, 382)	.02	277 (215, 362)	276 (214, 363)	.90	273 (214, 351)	280 (220, 371)	.28
*N*	545	109		33 665	6733		1200	240	

Test scores for reaction time and visual memory were compared. Only participants with no missing values for age, sex, and cognitive test scores for reaction time and visual memory are presented. Three participants are presented in both the cAMP LoF variant column and the G40S variant column as they had both variant types. A higher test score is indicative of lower cognitive function. Data are presented as median (first quartile, third quartile).

Abbreviations: GCGR, glucagon receptor; LoF, loss-of-function; T2D, type 2 diabetes.

#### Prevalence of all-cause dementia in individuals with variants of the GCGR

The frequency of all-cause dementia in individuals with a GCGR cAMP LoF mutation was 2.65% compared to 1.93% in nonvariant carrier controls, with a nonsignificant odds ratio (OR) of 1.39 [95% confidence interval (CI) 0.44-4. 373, *P* = .58] and the OR (1.59, 95% CI 0.49-5.13) remained nonsignificant after adjusting for age and sex (*P* = .45). In individuals with the G40S allele and individuals with a frameshift mutation of the GCGR, the frequencies of all-cause dementia were 1.72% and 1.20%, respectively, compared to 1.93% in controls. After adjusting for age and sex, there was, similarly, no significant difference in the OR between the GCGR G40S variant carriers (OR 0.90, 95% CI 0.74-1.08, *P* = .24) or frameshift variant carriers (OR 0.62, 95% CI 0.20-1.93, *P* = .35) compared to noncarrier controls (Table 4). When assessing the frequency of Alzheimer's disease alone, there were also no differences between the OR for nonvariant carrier controls and individuals with a cAMP LoF GCGR variant after adjusting for age and sex (OR 1.18, 95% CI 0.16-8.59, *P* = .87), or between nonvariant carrier controls and individuals with the G40S allele or frameshift variant (data not shown).

**Table 4.** Prevalent and cumulative all-cause dementia at end of follow-up (including everyone regardless of cognitive testing at baseline)

Dementia prevalence at follow-up	Nonvariant carrier controls	cAMP LoF variant	G40S variant	Frameshift variant
N	395 452	113	6924	249
Age at follow-up	73 (66, 78)	71 (65, 77)	73 (66, 78)	73 (66, 79)
Sex (female), %	54.0	59.3	55.0	57.8
T2D, %	8.6	6.2	8.6	7.2
All-cause dementia, n	7623	3	119	3
Prevalence, %	1.93 (7623/395 452)	2.65 (3/113)	1.72 (119/6924)	1.20 (3/249)
Odds for dementia	0.0197	0.0272	0.0175	0.0121
Odds ratio for dementia	1 (ref)	1.39 (0.44 to 4.37)	0.89 (0.74 to 1.07)	0.62 (0.20 to 1.93)
Age- and sex-adjusted odds ratio for dementia	1 (ref)	1.59 (0.49 to 5.13)	0.90 (0.74 to 1.08)	0.58 (0.18 to 1.82)

Descriptive data and dementia prevalence for individuals with a GCGR variant (cAMP LoF, G40S, or frameshift) and nonvariant carrier controls at follow-up (November 2023). Three participants have both the cAMP LoF variant and the G40S variant and are presented in both columns. Statistical differences were assessed by logistic regression analysis using nonvariant carrier controls as the reference and with and without adjusting for age and sex. Age is presented as median (first quartile, third quartile), and odds ratio is presented as odds ratio with 95% confidence interval.

Abbreviations: GCGR, glucagon receptor; LoF, loss-of-function; T2D, type 2 diabetes.

## Discussion

Here we show that the GCGR is expressed to a varying degree in cortical and subcortical regions of the human brain, including the frontal cortex and as expected in the hypothalamus [9, 14]. Antibody-dependent and -independent methods revealed GCGR localization in neurons but not astrocytes or microglia in the putamen, associated with motor control and coordination, and in the frontal cortex in Brodmann area 47, involved in cognitive functions including working memory, attention, and executive function. To our knowledge, the presence of the GCGR in the human frontal cortex and putamen has not previously been reported. The antibody-independent analyses were limited to the frontal cortex (to validate our antibody-dependent findings) as we aimed to assess the potential impact of GCGR expression in an area related to higher cognitive functions. The prevalence of all-cause dementia was not increased in individuals with a known or presumed GCGR LoF variant. However, lower cognitive ability, as assessed by 1 cognitive test, was shown for individuals with a cAMP LoF GCGR variant compared to age- and sex-matched nonvariant carrier controls. Lower cognitive ability—as assessed by the visual memory test—in individuals with a GCGR cAMP LoF variant indicates that impaired glucagon signaling in the brain may contribute to lower cognitive function.

We employed an extensively validated GCGR antibody [17]. Nonetheless, GCGR antibodies have limitations, which include unspecific binding properties, and may explain the low but detectable protein levels of the GCGR in mock-transfected HEK-293 cells. However, we found negative Western blotting of the neurohypophysis in line with no detectable mRNA levels [16]. Importantly, we show GCGR mRNA in frontal cortex sections, thus validating our antibody-dependent findings in the frontal cortex. Nevertheless, these findings should be repeated by others.

In our study, we used postmortem brain tissue, and it is important to stress this as a potential limitation due to factors such as high age and postmortem to preservation interval. Therefore, the sample size used in this study may be underpowered to determine the scale of the expression differences across brain areas.

Dual [glucagon/glucagon-like peptide 1 (GLP-1)] and triple (glucagon/GLP-1/glucose-dependent insulinotropic polypeptide) agonist therapies that include glucagon receptor activity are currently being investigated for the purpose of body weight loss in phase 3 studies. GLP-1 is processed from the same prohormone as glucagon and has been shown to have neuroprotective properties in preclinical [28-31] and clinical studies [32, 33]. Here we show that impaired GCGR signaling may be associated with diminished cognitive function as evaluated by cognitive tests, similar to a previous study [34], highlighting the possibility that glucagon may also have neuroprotective properties. Conversely, hypothalamic GCGR signaling has previously been shown to suppress hepatic glucose production [14] and feeding [35]. Impaired GCGR signaling could disrupt these regulatory axes, thus contributing to hyperglycemia and insulin resistance—2 conditions associated with cognitive decline and dementia—as well as metabolic dysregulation. However, individuals with the cAMP LoF variant had lower cognitive ability despite a lower prevalance of T2D as compared to nonvariant carrier age- and sex-matched controls, which could suggest that impairments in central GCGR signaling harm cognitive function independently of diabetes Therapeutic interventions activating GCGR signaling remain to be assessed in connection with their impact on cognitive health.

All-cause dementia includes many etiologies, whereas central insulin resistance has been linked to Alzheimer's disease specifically. Nevertheless, we found no additional risk of Alzheimer's disease with impaired GCGR signaling. This finding may be due to the small number of carriers, the heterozygosity of the mutation for the cAMP LoF mutation, or perhaps a dementia underdiagnosis or presentation at a more advanced age.

Glucagon signals through its G-protein coupled receptor, coupled to either Gαq or Gαs. GCGR Gαs signaling activates adenylate cyclase, which leads to the formation of cAMP. However, all the GCGR cAMP LoF variants (except V368M) also have a lower maximal β-arrestin-2 recruitment [15], and we can therefore not exclude the partial effect of impaired β-arrestin-2 recruitment on the activity of GCGR signaling. Conversely, lowered cAMP accumulation may lead to a reduced phosphorylation of cAMP response element-binding protein (CREB), a transcription factor crucial for neurogenesis and synaptic plasticity [36]. This could impair the expression of genes involved in neuronal growth, dendritic spine formation, or synaptic strength [37]—processes that are also disrupted in Alzheimer's disease pathophysiology. The GCGR G40S allele, which impairs β-arrestin-1 recruitment [15], is associated with T2D, hypertension, altered renal sodium handling, and increased central adiposity in men [38-41], highlighting that impaired GCGR signaling is detrimental despite its key role in promoting hepatic glucose production.

We have previously reported hepatic glucagon resistance in individuals with T2D and steatotic liver disease. Excessive mTORC signaling has been observed in neurodegenerative diseases including Alzheimer's and Parkinson's disease and may contribute to neuronal dysfunction and degeneration [42]. Glucagon suppresses hepatic mTORC activation, and 1 potential mechanism by which impaired glucagon signaling could contribute to cognitive impairment is through brain glucagon resistance, leading to increased mTORC activity. However, this requires mechanistic validation.

To conclude, the GCGR is expressed in various different areas throughout the brain, including neurons of the frontal cortex. Carriers of known or presumed GCGR LoF variants do not have an increased risk of all-cause dementia or Alzheimer's disease, but individuals with a cAMP LoF GCGR variant have lower cognitive function as evaluated by 1 population-scale cognitive test. Collectively, our data suggest a potential association between compromised central GCGR signaling (lowered cAMP accumulation) and cognitive function. However, these findings warrant further investigation.

## Data Availability

Original data generated and analyzed during this study are included in this published article or in the data repositories listed in References. All UK Biobank data from this study can be acquired from the UK Biobank. Coding is available through https://github.com/nicwin98/GCGR-Neuro.
